# The effects of common-sense model interventions on cancer patients: A systematic review

**DOI:** 10.1097/MD.0000000000037777

**Published:** 2024-04-26

**Authors:** Xue Gu, Xia Shen, Jun-Rui Zhou, Jiang-Hui Chu, Lei Jiang

**Affiliations:** a Wuxi School of Medicine, Jiangnan University, Wuxi, China; b Department of Cardiothoracic Surgery, Affiliated Hospital of Jiangnan University, Wuxi, China; c Department of Radiology, Huadong Sanatorium, Wuxi, China.

**Keywords:** cancer, common-sense model, illness perception, intervention, review

## Abstract

**Background:**

From the time of new diagnosis to treatment, cancer patients experience a variety of health problems that can affect the patient’s health outcomes. Individuals with cancer are being given increasing responsibility for the self-management of their health and illness. The self-regulating common-sense model (CSM) is effective in patients’ disease management. This article briefly introduces the common-sense model intervention, in which patients with cancer are affected by these interventions, what they are about, and what effects they have.

**Methods:**

The authors systematically review evidence for the common-sense model of self-regulation for cancer using Preferred Reporting Items for Systematic reviews and Meta-Analyses (PRISMA) guidelines. Based on a comprehensive literature search, we searched the Cochrane Library, PsycINFO, Embase, PubMed, Medline, CINAHL, CNKI, and WanFang databases. The included studies underwent a quality assessment using the Effective Public Health Practice Project (EPHPP).

**Results:**

Eleven empirical studies illustrated the aspects of common-sense model interventions for cancer patients. It is concluded that common-sense model intervention has an effect on symptoms in cancer treatment, behavior, and quality of life, but more studies are needed to verify the use of common-sense model intervention to explore in patients with different cancers. The systematic review summarized a four-point paradigm about intervention content, including assessing the current situation, setting goals, having a disease education and psychological adjustment, and getting feedback for further response. However, the application of intervention requires specific analysis of patient behavior and outcomes.

**Conclusion:**

Common-sense model interventions are beneficial for the self-management of cancer patients; however, more intervention studies are needed to specify the cognitive, emotional, and coping styles of people with a particular cancer.

## 1. Introduction

Cancer is a major cause of morbidity and mortality, worldwide. The burden is projected to increase, with 13 million cancer-related deaths occurring annually by 2023.^[1]^ Efforts need to be made by all countries to effectively evaluate, plan, and implement cancer control initiatives.^[2]^ Reducing environmental pollution, improving dietary interventions, promoting physical activity, and early screening can prevent cancer. Primary prevention is a particularly effective way to fight cancer.^[3]^ After diagnosis, patients undergo a wide range of cancer treatments, including surgery, chemotherapy and radiotherapy, to improve survival.^[4]^ Improvements in management measures to prevent risk factors, early screening policies, and the search for reliable and accurate diagnosis methods have all played a role in cancer control and management. Additionally, changes in treatment technology, such as the widespread use of less invasive thoracoscopy and innovations in nanotechnology, have contributed significantly.

With the increasing number of cancer survivors, the traditional care model led by cancer specialists (medical oncology, radiation oncologists), is becoming less capable of providing long-term care, leading to a heavy. Three alternative models of primary-care providers exist: care shared between oncology specialists and primary-care providers, and care led by oncology nurses, each offering corresponding benefits for cancer survivors. Choosing the most appropriate model of care for each patient depends on patient-level factors such as risk of long-term effects, late effects, personal desire and the ability to self-manage, as well as local services and healthcare policies. In the process of medical care, it is essential to respect the wishes and needs of cancer patients, which also shows that patients themselves take more and more responsibility for treatment and care.^[5]^ From the time of new diagnosis to treatment, cancer patients experience a variety of health problems that can affect the patient’s health outcomes. Individuals with cancer are being given increasing responsibility for the self-management of their health and illness.^[6]^ Cancer patients’ perception of the symptoms generated during treatment and their knowledge of the disease and emotional support determine their coping behavior and self-management.^[7]^

The self-regulating common-sense model (CSM) is effective in patients’ disease management. It helps patients recognize the factors that threaten their health and develop interventions to reduce the disease burden by addressing for disease perception. CSM has a history of more than 50 years.^[8]^ The model posits that stimuli activate either current or future health threats, leading individuals to produce cognitive and emotional responses to the disease, which in turn prompt behavioral measures and result in various outcomes.^[9]^ The common-sense model of self-regulation has been applied to patients with chronic diseases, heart disease, diabetes, mental illness, and post-surgery condition.^[10–12]^ It affects patients’ psychology and cognition, and improves health behaviors such as patient compliance, as well health outcomes including quality of life, depression, and physical function.^[13–15]^ We found that many qualitative and quantitative studies on disease characteristics, and intervention are still to be explored.^[16]^ For patients with cancer, we examined the effects of disease representation on coping behavior and disease outcomes.^[17]^ We have found some intervention studies on cancer patients based on the self-regulating common-sense model.^[18,19]^ However, the question remains: how do researchers then apply the self-regulating common-sense model-based intervention to cancer patients, and can the effects of the intervention? Therefore, we would like to conduct this systematic review to determine to what extent and how CSM is used in patients with cancer to provide a basis for its future application in clinical cancer patients.

The research questions addressed are as follows: How does the common-sense model intervention affect cancer patients? What are the specific aspects of these interventions, and what effects do they have?

### 1.1. Patient involvement

This review attempts to provide reference for the intervention content for cancer patients to use the self-regulating common-sense model to improve their own health status in the future. Our team will conduct future research on interventions based on the self-regulating common-sense model to explore the disease perception and lung function exercise compliance of patients with lung cancer after thoracoscopic surgery.

## 2. Method

As a systematic review, this study did not necessitate an institutional review board or research ethics committee approval. The systematic review is an analysis and summary of the published literature.

### 2.1. Search methods

**Figure 1.**
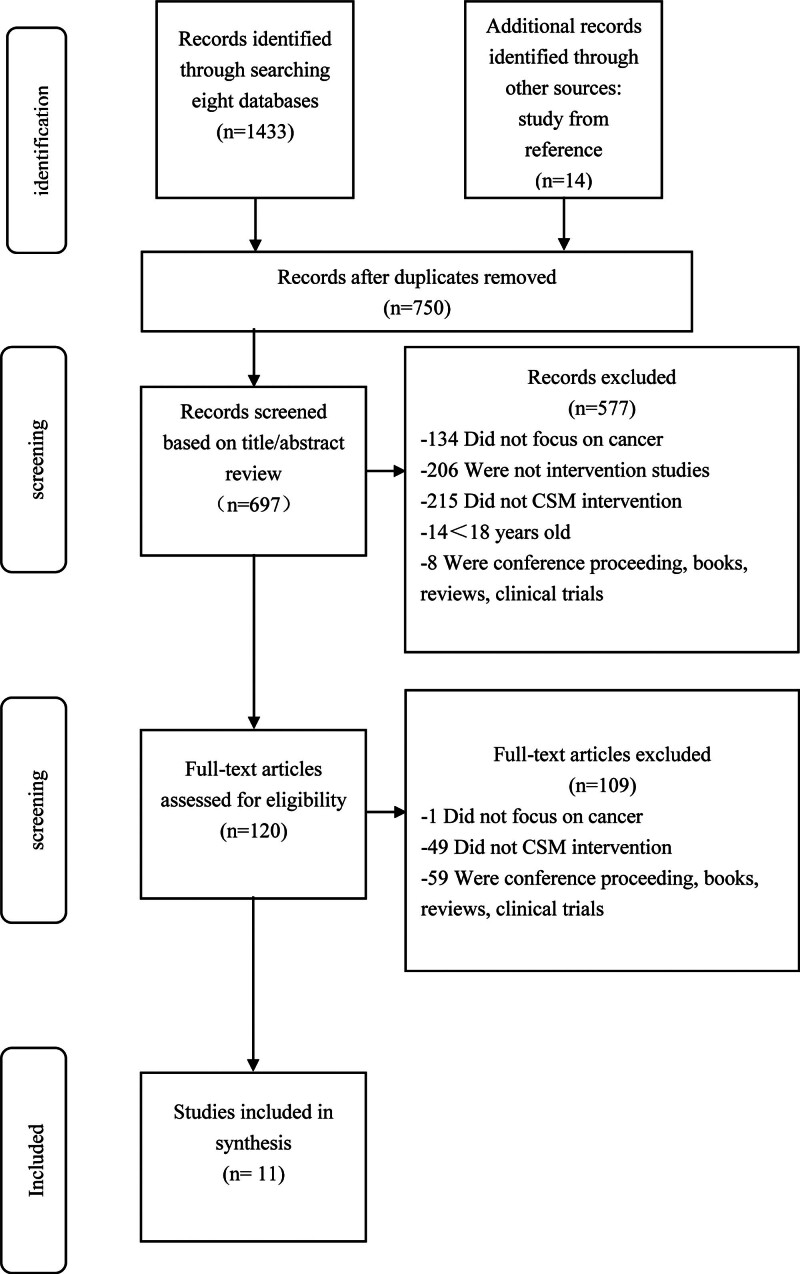
PRISMA diagram of review selection process.

This systematic review was performed in accordance with the PRISMA (Preferred Reporting Items for Systematic Reviews and Meta-Analyses) guidelines.^[20]^ We systematically searched the literature in 6 English databases (Cochrane Library, PsycINFO, Embase, PubMed, Medline, CINAHL) and two Chinese databases (CNKI and WanFang Data), covering the period from each database’s initiation to February 2023. The search key terms “cancer” or “tumor” or “neoplasm” or “oncology” AND “illness perception” or “illness representation” or “illness cognition” or “common-sense model” or “self-regulation” or “self-regulatory” AND “randomized controlled trial” or “intervention” or “random allocation” or “clinical trial” or “random*” or “controlled” or “trial” or “rct” or “placebo” or “controlled clinical trial” used for searching English databases. “癌症” or ”肿瘤” or “癌” AND “疾病感知” or “疾病认知” or “自我调节常识模型” or “常识模型” or “自我调节” AND “干预” or “随机对照” or “对照” as key words to search Chinese databases for eligible studies. CNKI and WanFang are two authoritative Chinese databases that include a large number of Chinese articles. In addition, we limited our search to literature in English and Chinese languages. A manual search of the reference lists of the included studies and relevant authors was conducted to identify additional eligible studies. The retrieved literature was entered into the Endnote database and duplicates were deleted. Figure 1 shows the flow chart.

Because some theory-based intervention articles did not include the theoretical name in the title and abstract, we searched for words with similar disease perception to search for more articles.

### 2.2. Selection criteria for identifying articles

In this systematic review, we selected these studies based on 2 criteria: they involved cancer patients at different stages, and applied the self-regulating common-sense model intervention.

The exclusion criteria included: patients without relevant treatments, such as cancer screening for healthy individuals, The patient is less than 18 years old (Cancer patients are adults), studies not involving intervention with the CSM, and studies that were reviews, conferences, trial registries, protocols, and incomplete literature.

### 2.3. Data extraction and synthesis

The full extraction and synthesis of important information regarding the included studies are presented in 2 tables. Table 1 describe the author of the included articles, the year, the country, the purpose of the study, the type of design, the characteristics of the participants in the study, the theory used in the intervention. The content and dosage of the intervention, the implementer, and the way in which the intervention was used in Table 2. We extracted and synthesized the outcome measurements, measuring time, and study results, and analyzed the feasibility and acceptability in Table 3.

**Table 1:** Characteristics of included studies.

Author (Year) Country [reference]	Study aims	Study design	Cancer diagnosis (Stages); characteristic and no. of participants
Maryam Abedini et al (2019) Iran^[29]^	To investigate whether the Self-Regulation Model could improve sexual satisfaction for women diagnosed with breast cancer.	A randomized controlled trial	Inclusion criteria: women, breast cancer (various stages), undergoing treatment (chemotherapy or combination therapy); 18-55;married; the ability to communicate; Exclusion criteria: had a history of sexual disorders prior to the cancer diagnosis (i.e., vaginismus, intercourse pain), acute mental illness (i.e., depression and/or severe anxiety), physical illnesses (i.e., heart disease, pulmonary disease, skeletal and/or muscular diseases), or a history of previously receiving psychotherapy or counseling.
Nicole C Allard et al (2007) Canada^[32]^	To determine the efficacy of a nursing intervention based on self-regulation theory known as the Attentional Focus and Symptom Management Intervention (AFSMI) in enhancing physical and emotional well-being in women who underwent day surgery for breast cancer.	A randomized clinical block trial	Inclusion criteria: women, newly diagnosed with breast cancer(primary or a suspected lesion), undergo their first breast surgery(with or without axillary node dissection), >18 years, the ability to communicate, had no hearing impairment, had a phone at home. Exclusion criteria: to render the sample of women homogeneous with respect to surgical procedure, morbidity, and prognosis.
Amy E. Richardson et al (2017) New Zealand^[26]^	To investigate the effectiveness of a brief self-regulatory intervention (targeting illness perceptions and coping) at improving HNC patient health-related quality of life (HRQL).	A randomized controlled trial	Inclusion criteria: Diagnosed with a primary epithelial head and neck cancer(carcinoma in the pharynx, larynx, oral cavity, sinonasal cavity), or metastatic skin cancer in the head and neck region within 3 weeks prior to their clinic attendance, a treatment plan of one or more treatments(surgery, radiotherapy, chemotherapy) and their caregivers (spouse, family member, close friend), 18-90 years old, can communicate; Exclusion criteria: severe substance dependence, active psychosis, cognitive impairment, or significant physical disability, those to be treated with palliative intent
Sandra Ward et al (2000) USA^[22]^	A nursing intervention to address these “‘patient-related barriers’” was developed based on Johnson’s self-regulation theory. The purpose of this pilot study was to determine whether provision of individually tailored sensory and coping information about analgesic side effects and specific information to counter misconceptions would enhance pain management in a sample of 43 women with gynecologic cancers.	A randomized controlled trial	Inclusion criteria: women, metastatic or progressive gynecologic cancer, had experienced cancer-related pain in the last 2 weeks, diagnosis of metastatic or progressive gynecologic cancer with 2 weeks pain experience Exclusion criteria: patients were not approached to participate in this study on their first visit to clinic, or on a day they learned of failed treatment, disease progression, or on the first day of a new treatment.
Heidi Scharf Donovan et al (2001) USA^[19]^	To describe the theoretical basis for a representational approach (Leventhal’s common sense model (CSM)) to patient education and the application of this approach to the development, implementation, and preliminary evaluation of a representational intervention for pain management	A randomized clinical trial	Inclusion criteria: a diagnosis of progressive or metastatic cancer, a worst pain in the last 2 weeks commonly breast, colorectal, pancreatic cancer
Nicole C Allard et al (2016) Canada^[27]^	The study assessed whether a nursing intervention based on self-regulation theory, the Attentional Focus and Symptom Management Intervention (AFSMI), could help women who underwent day surgery for breast cancer to achieve better pain management and decreased emotional distress.	A randomized controlled trial	Inclusion criteria: women, newly diagnosed with breast cancer(primary or suspected lesion), undergo lumpectomy on a day-surgery basis, can communicate, over the age of 18 years, had no hearing impairment, had a phone at home; Exclusion criteria: previous with cancer and major psychiatric problems
Elizabeth A Grunfeld et al (2018) UK^[28]^	To assess the feasibility and acceptability of a theoretically led workbook intervention designed to support patients with cancer returning to work	A parallel-group randomized controlled trial	Inclusion criteria: received a diagnosis breast, gynecological, prostate or colorectal cancer, had not been classified as having metastatic disease or recurrence, at least 2 weeks post-treatment initiation, aged 18–70 years, working at the time of diagnosis; and not working at time of recruitment but intended to return to work
Kim Y et al (2002) USA^[23]^	To examine the effects of an informational intervention on the severity of side effects resulting from radiation therapy for prostate cancer.	A randomized clinical trial	Inclusion criteria: receiving RT as curative treatment for localized prostate cancer as outpatients, having no previous or concurrent cancer diagnosis (except basal cell skin cancer), being able to speak and read English, having no history of mental illness or alcoholism, being capable of meeting daily basic needs independently (Karnofsky Performance status of at least 80%), and being 18 years of age or older
Yanhong Yao et al (2020) China^[24]^	To explore the application method and effect of nursing program based on self-regulation theory combined with HBM health education in patients with intracranial tumor surgery	A randomized controlled trial	Inclusion criteria: over 18 years old, Perform surgery for intracranial tumors. Exclusion criteria: Patients with a history of mental illness, hearing and vision impairment, severe damage to liver and kidney function, and other malignant tumors
Yiping Yuan et al (2022) China^[25]^	To explore the effect of self-regulation theory combined with special nursing on patients with colon cancer implanted in intravenous infusion port	A randomized controlled trial	Inclusion criteria: ≥30 years old, Pathological diagnosis of colorectal cancer, An indwelling port for implantable intravenous infusion. Exclusion criteria: Heart or other organic disease, serious mental disorder
Di Gu et al (2020) China^[30]^	To investigate the effect of self-regulation nursing program on the compliance of patients with non-small cell lung cancer (NSCLC) treated with intravenous combined with chemotherapy	A randomized controlled trial	Inclusion criteria: The diagnosis of NSCLC was confirmed by histopathology, all patients received intravenous Avastin and chemotherapy. Exclusion criteria: Have a history of allergies to the drugs they are taking, targeted drug therapy and other regimens were used, combined with mental disorders, cardiovascular and cerebrovascular diseases, senile dementia, Life expectancy < 1 year

**Table 2:** Details of interventions of included studies.

Author (Year) Country [reference]	Intervention content and dosage	Delivery format (Who and How)
Maryam Abedini et al (2019) Iran^[29]^	Intervention: Before beginning the psychoeducation intervention, the goals are list that are their main concerns regarding their cancer diagnosis and focus on the main concern for future interventions. 1. intervention content: psychoeducation intervention (5 perceptions of breast cancer about consequences (impact on life of symptoms), time (duration of treatment recovery), control (coping with the consequences of treatment or treatment and coping with emotions - concern, emotional dimension), identity (perception of symptoms), coherence (understanding disease)). coping: practice skill training exercise; manage negative emotions about cancer in self-control exercises; encourage to engage in positive behaviors, spousal support is a way; Participants were encouraged with mindfulness and physical activity; Share cancer experiences with women(Positive perspectives Transfer disease perspectives); The pamphlet contains information on cancer and ways to cope with sexual satisfaction. 2. Summarize the first lesson questions. Address currently recognized issues, find the causes of hypoactive sexual desire disorder and chemotherapy side effects, other patients shared experiences of sexual intercourse, such as sexual intercourse is reduced due to lack of intimacy or fear of infection. 3. Assess sexual satisfaction again. Coping: whether they had solutions for improving sexual intercourse. Time: 3 sessions lasted for 60-90 minutes	Therapist session
Nicole C Allard et al (2007) Canada^[32]^	Intervention: Using the interview guide and a follow-up sheet, the investigator assessed each woman’s symptoms by asking her to identify and describe each symptom in concrete, objective terms. The actions taken by each woman to manage each symptom and the effectiveness of her actions in relieving symptoms were rated using a 5-point Likert scaled ranging from 1 (not effective) to 5 (very effective). Actions that women felt were effective in managing their symptoms were encouraged by the intervener. If their actions were ineffective, women were encouraged to find other potentially helpful actions. The investigator suggested new or additional self-care strategies when requested. During telephone contact, the intervener acknowledged any feelings or emotions women expressed. Time: 2 sessions, 3–4 days and 10–11 days after surgery. the length of the telephone contact was not limited because was individualized	Investigators telephone
Amy E. Richardson et al (2017) New Zealand^[26]^	Intervention: psychological interventions delivered early after diagnosis brief self-regulatory intervention (targeting illness perceptions and coping). 1. understand accurate illness perception; 2. managed distress ways, symptoms, treatment side effect (coping targeted), specify when, where, how, and with whom together. 3. evaluated the effectiveness and prepared patients for following the completion treatment. educational manual: Head and neck cancers (types, causes, symptoms and stages); Treatment (surgery, chemotherapy and radiotherapy); Side effects; coping ways; Managing relationships (with family, children, friends, and healthcare professionals); And the support provided by the community. Time: 3 sessions, 60-min each session. The first time was taking place prior to treatment commencement, the second was the beginning of treatment, the third was the end of treatment, 3 weeks after the last session 30 min follow-up phone.	Health psychologist face to face session (hospital or home)
Sandra Ward et al (2000) USA^[22]^	Intervention: 1. provide knowledge and coping information about analgesic side effects. 2. Inform patients that misconceptions would enhance pain management. 3. information intervention: individually tailored information about concerns barriers (reference to a booklet about the 8 patient-related barriers) and side effect management (sensory and coping information regarding analgesic side effects) The way to learn is, first, encourage to concern about addiction and constipation and nausea side effect, then hints on how to manage them. then, resolve patients’ problems. Time: Two sessions, 25 min each session.	Nurse clarification phone call
Heidi Scharf Donovan et al (2001) USA^[19]^	Intervention: the intervention begin with an assessment of person’s representation of cancer pain (5 dimensions) 1. Have a representational assessment that encouraged patient to describe illness representations along the 5 demonstrations (identity, cause, timeline, consequences, and cure or control) 2. encourage patient to think about what experiences led to misconceptions 3. patients discussed limitations of misconceptions: the reason of maintaining misconceptions, links between misconceptions and consequences of acting on them. 4. present credible information to replace current misconceptions 5. Have a summary to discuss benefits associated with acting on new information. Time: Five sessions, each education time is the range of 20-72 min.	APNs advanced practice nurse counseling interview
Nicole C Allard et al (2016) Canada^[27]^	Intervention: 1. patients managed each symptom. 2. Nurse assessed actions that the effectiveness of these actions in relieving their symptoms were explored using a 5-point Likert scale ranging from 1 to 5 very effective. 3. If patients felt effective, nurse were encouraged, or opposite, nurse suggested new or additional self-care strategies. 4. the intervener acknowledged any feelings and emotions patients expressed. Time: 3-4 days and 10-11days after surgery. The intervener made one phone call a week for a total of two telephone intervention sessions for each woman. The length of the telephone contact was a function of the number of symptoms experienced or of any other concerns that the woman was willing to discuss.	Oncology nurse telephone interventions
Elizabeth A Grunfeld et al (2018) UK^[28]^	Intervention: the workplan package is a 4-week guided workbook intervention consisting of structured sections and activities to provide guidance and support for patient. 1. Patients think about illness and treatment (based around the illness perceptions), and explore the impact of cancer and treatment to function in the workplace. Nurse examined participants’ emotional reactions to treatment and support/strategies to manage these. 2. focus on setting and achieving goals (based on goal theory) including the goal-setting process, identify and overcome barriers and support themselves. 3. Build confidence. 4. develop an action plan for returning to work and outline, deal with difficult. Time: 4-week guided workbook intervention and 120 min per week. work through chapters in turn during each week of the intervention period.	Research nurse/ research assistants interview telephone face to face; workbook intervention-workplan package
Kim Y et al (2002) USA^[23]^	Intervention: Patients listened to brief tape-recorded messages in the clinic before their first and fifth RT, so two interventions in total, respectively 4 min and 8 min. The first message contained an explanation of RT adapted from pamphlet(Radiation Therapy and You, the different types of RT and the use of high-energy X-rays, the type, location, and size of the tumor). The second message described the services available at the treatment facility, the roles of staff, community services available in the area. 1. the first message described the procedures that would be encountered during simulation, the clinical set-up, and what would occur at the first treatment. 2. the second message is that would be encountered during the succeeding weeks of treatment. Focusing on physical and sensory experiences and environmental, temporal characteristics when the machine moved and specific side. Clinic personnel answered all questions patients had concerning their treatments. Time: two session (the first RT and the fifth RT), RT = radiation therapy	Research staff/clinic personnel the tape-record message/face to face
Yanhong Yao et al (2020) China^[24]^	Intervention: 1. Assess patients’ disease perception, give an admission health education to form a nursing concept upon admission; 2. Inform patients of measures to prevent adverse reactions after surgery to enhance self-efficacy and health beliefs about intracranial tumor disease. 3. Before patients were discharge, the basic situation of patients and the knowledge of post-operative prevention were assessed, the dosage and methods of medication and precautions were informed, the importance of continuous medication was distributed, and the knowledge manual of intracranial tumor was distributed 4.After discharge, In the first month, 1 week after discharge to remind the phone to read the manual; Help patients to understand their own condition and the symptoms of disease aggravation and the reasons for recurrence, consult the medication compliance and whether there are discomfort symptoms, and choose the appropriate medication reminder plan according to the patient’s situation, 15-30min, once a week. In the second month, the disease control situation was discussed, psychological counseling was carried out, successful cases of treatment were introduced, and finally the compliance was consulted, 15–30 min, once a week. In the third month, intensive intervention were for existing problems, 15–30 min, twice a week. Time: More than once a week for 3 months	Attending physician, clinical nurse face to face, handbook, telephone
Yiping Yuan et al (2022) China^[25]^	Intervention: 1. Firstly, the patients were investigated by questionnaire or orally to understand the status of intravenous infusion port implantation, diet, sleep, emotional symptoms, current disease cognition, treatment effect, and recovery confidence. 2. Have a cognitive intervention, focusing on health education for patients and their families, including colon cancer and infusion port information. explain self-regulation theory and introduce relevant successful cases in response to emotional questions; develop measures to set appropriate goals for daily activities and disease observation and relief of adverse mood; make good records and adjust goals and behaviors that occur in the execution. 3. Have a psychological nursing to relieve bad emotions. encourage patients to consult and support each other in the discharge care of we-chat group. Time: The number of interventions and the duration of each intervention were not specified	Nurse face to face, We-chat group
Di Gu et al (2020) China^[30]^	Intervention: 1. Develop a self-regulating nursing plan and find out the current problems of patients according to the information of self-efficacy, psychological resilience and targeted therapy in the interview. 2. Have a pretreatment health education, distribute intravenous combined with chemotherapy treatment guidelines, introduce treatment precautions and benefits to understand the treatment for patients, introduce the environment to increase patients’ psychological adaptability. 3. Have a care during treatment, explain the toxic and side effects of drugs, reduce patients’ fear and worry about drugs, improve disease management ability, encourage to use protein and digestible food during medication, inform patients of their condition with the consent of patients and their families, and encourage them to actively face the disease. 4. During the follow-up period, urge patient to record regular medication plan card, push message in we-chat public platform to build confidence Time: The number of interventions and the duration of each intervention were not specified	Nurse face to face, telephone

**Table 3:** Outcomes of included studies.

Author (year) Country [reference]	Outcome measurements (Measurement intervals)	Program evaluation outcomes	Intervention effects (*P* < .05 indicates statistical significance)
Maryam Abedini et al (2019) Iran^[29]^	Measure outcomes: index of sexual satisfaction (ISS) Time: Pre-intervention measurement (upon referral to the hospital); Post-intervention measurement (They were measured after 1 month of intervention,2,3 months following their referral to the hospital)	Feasibility: Recruitment rate, unspecified; Completion rate: Attrition rate 8%, control n = 40-36; intervention n = 40-37 Acceptability: agreement: unmeasured limitations: Intervention is recommended when the patient is in a better state	Outcomes: The intervention group about satisfaction score had a positive increasing trend but the control had a deteriorating trend. There was statistical difference between the 2 groups (*P* < .05). 1 months after the intervention. There was statistical difference between the 2 groups (*P* = .001).
Nicole C Allard et al (2007) Canada^[32]^	Measure outcomes: symptom impact profile (SIP), measured a patient’s progress and the recreation pastimes home management reflect functional status; profile of mood states (POMS), measured emotional distress. Time: Pre-intervention measurement (T1 pretest); post-intervention measurement (T2 one week following the intervention session; T3 one week following the second intervention session)	Feasibility: Recruitment rate, 64% (1-65/182); Completion rate: attrition rate 0%, control n = 56 intervention n = 61 Acceptability: agreements: unmeasured; limitations: The content of intervention is not yet determined Indefinite frequency, intensity, duration; the sample is the lack of diversity.	Outcomes: There was statistical difference between the 2 group about Function status (home management disruption); Comparisons showed a significant difference between the experimental and control group at T2 (t=–2.20; *P* = .03) on emotional distress scores.
Amy E. Richardson et al (2017) New Zealand^[26]^	Measure outcomes: functional assessment of cancer therapy-head and neck (HRQL): measure physical, social, emotional, functional wellbeing designed to assess head and neck specific wellbeing; general health questionnaire assess symptoms of psychological distress (GHQ-12); the brief illness perception questionnaire (IPQ); Satisfaction with intervention about 4 open-ended questions (general satisfaction with sessions received, aspects of the intervention that were considered most beneficial, aspects of the intervention that could be improved, and whether the intervention could be recommended to other patients diagnosed with HNC.) Time: Pre-intervention measurement (baseline); post-intervention measurement (again 3 and 6 months)	Feasibility: Recruitment rate, 46% (64/139); Completion rate: attrition rate 13% control n = 31-25 intervention n = 31-29 Acceptability: 93% reported being satisfied; limitations: no active control group; not restricted to patients experiencing distress.	Outcomes: Compared to the control group, patients who received the intervention had increased treatment control perceptions at 3 months. There was statistical difference between the 2 group (*P* = .01). and increased social quality of life at 6 months. The intervention was particularly helpful for patients exhibiting distress at baseline.
Sandra Ward et al (2000) USA^[22]^	Measure outcomes: Illness perception: barriers questionnaire; pain management index; medication side effect checklist (MSEC); Outcomes: functional assessment cancer therapy-general (FACT-G). participant satisfaction as measured by information evaluation: the information intervention in terms of helping them to talk more openly about pain, be less concerned about addiction, feel more comfortable taking pain medications, response options were no, not sure, yes. Time: pre-intervention (baseline) post-intervention (1 month post-test and 2 months follow-up)	Feasibility: Recruitment rate, 56% (43/77) Completion rate: attrition rate 19%, 43-33 completed 1 month post-test measure-27 completed 2 months follow-up measures. 25 women completed measures at all 3 time points Acceptability: 86% believed the information would be helpful to them; limitations: the potential source of contamination is clinicians change their behavior; the greatest need patients don’t participant intervention; Long-term observation is needed	Outcomes: All women reported a decrease in barriers between baseline and 2-month follow-up; all subjects experienced a decrease in pain interference with life scores between baseline and 1-month post-test; and there was a significant shift of women from unacceptable pain management at baseline to acceptable pain management at 1-month post-test.
Heidi Scharf Donovan et al (2001) USA^[19]^	Measure outcomes: Qualitative investigation of pain management Time: pre-intervention (baseline) post-intervention (immediately after the intervention and 2 months later)	Feasibility: Recruitment rate, unspecified Completion rate: attrition rate 7%, 61-57 completed follow-up education. Acceptability: 94% participants said the information would be helpful to them.	Outcomes: 39 of the 47 participants who returned follow-up questionnaires said the intervention had changed the way they think about pain medication; 40 (85%) said they were more confident using pain medication; 38 (80%) indicated more confidence talking with their physicians or nurses about pain; 32 (68%) reported that their pain was better managed; 27 (57%) were better able to manage the side effects of pain medications; and 27 (57%) reported making changes in the way they managed their pain as a result of the intervention.
Nicole C Allard et al (2016) Canada^[27]^	Measure outcomes: Multidimensional fatigue inventory (MFI); present pain intensity (PPI); severity of insomnia index; symptom impact profile (home management); profile of mood state subscales and associated total score Time: pre-intervention (post-operate 2-3 days baseline) post-intervention (post-operate 9-10, 17-18days)	Feasibility: Recruitment rate, 64.28% (117/182); Completion rate: attrition rate 0%, 61 experimental group, 56 usual care group. Acceptability: agreements: unspecified limitation: the AFSMI was not effective in reducing pain, fatigue, and insomnia experienced following breast surgery. The patient currently has good symptom management, as healthcare professionals need to be aware of the presence of symptom clusters; the symptoms were resolved rather quickly during the early recovery period. Need objective indicator measurement.	Outcomes: Results showed significant differences between the experimental and control group at post-test on home management at t3 (*P* = .01), confusion t2 (*P* < .05), emotional distress t2 (*P* = .03).
Elizabeth A Grunfeld et al (2018) UK^[28]^	Measure outcomes: Interview focused on: beliefs about work and cancer; experience of employment and work values; ways in which returning to work could be supported; and expectations of the Work-Plan intervention. Twelve-month interviews explored: beliefs about cancer and work and how these were challenged over the preceding year, general perceptions of the trial and the personal return to work process of each individual. Time: pre-intervention(baseline) post-intervention (4-week postintervention or 4-week, 6-month, 12-month (follow-ups)	Feasibility: Recruitment rate, 21% (68/324); Completion rate: attrition rate 19% (intervention 34-26 usual 24-21) Acceptability: agreements: the overall participants enjoyed taking part in the intervention, the booklet was seen as convenient, simple to transport and could easily be shared with others. limitation: the sample size was underpowered; an economic evaluation was not suitable; the study did not test the fidelity of the intervention	Outcomes: At 6-month follow-up, 30% of the usual care group had returned to full-time or part-time work (including phased return to work) compared with 43% of the intervention group. At 12 months, the percentages were 47% (usual care) and 68% (intervention). secondary outcome measures, the intervention group reported less anxiety and depression-related symptoms.
Kim Y et al (2002) USA^[23]^	Measure outcomes: Patients are rated the severity on a 5-point Likert scale about diarrhea, fatigue, skin changes, sleep disrupting, urinary problem; The profile of Mood states (POMS) measure the tension-anxiety, anger-hostility, depression-dejection. Time: post-intervention (the second RT; the last RT)	Feasibility: Recruitment rate, 83% (152/184) Completion rate: attrition rate 0%, 77 experimental group, 75 usual care group. Acceptability: agreements: unspecified limitation: Don’t have baseline information about symptom severity; Have an advice to use standardized scales/devices.	Outcomes: The results showed that patients in the informational intervention group reported significantly fewer problems with sleep and less fatigue (marginally significant) than those in the comparison group.
Yanhong Yao et al (2020) China^[24]^	Measure outcomes: Self-made compliance questionnaire; LOTCA Cognitive function rating scale(Loewenstein); Incidence of postoperative adverse reactions: pulmonary infection, elevated intracranial pressure, incision infection, hyponatremia, heart failure; Self-made satisfaction questionnaire Time: pre-intervention and post-intervention	Feasibility: Recruitment rate, unspecified Complete rate: attrition rate 0% (intervention n = 36 usual n = 34) Acceptability: agreements: the nursing satisfaction was higher.	Outcome: The nursing compliance of the observation group was higher than that of the control group. There was statistical difference between the 2 groups (*P* < .05). the scores of LOTCA and MMSE in the observation group were higher than those in the control group after nursing. There was statistical difference between the 2 groups (*P* < .05).The incidence of adverse reactions in the observation group was lower than that in the control group. There was statistical difference between the 2 groups (*P* < .05). The nursing satisfaction was higher than that in the control group. There was statistical difference between the two groups (*P* < .05)
Yiping Yuan et al (2022) China^[25]^	Measure outcomes: HADS Hospital Anxiety and Depression Scale; AIS Athens Insomnia Scale; Fop-Q-SF Simplified scale of fear of disease progression in Chinese cancer patients; SUPPH Cancer self-management efficacy Scale; QLACS Quality of Life Scale for cancer Survivors Time: pre-intervention and post-intervention	Feasibility: Recruitment rate, unspecified Complete rate: attrition rate 0% (intervention n = 43 usual n = 45) Acceptability: agreements: unspecified	Outcomes: Observation group after intervention hospital anxiety and depression scale (HADS), the scores of insomnia scale (AIS) and simplified fear of disease progression Scale (FOP-Q-SF) in Chinese Cancer patients were lower than those in control group. There was statistical difference between the 2 groups (*P* < .01); The incidence of postoperative complications, the scores of financial problems, recurrence concerns, image problems and family stress in cancer quality of life scale (QLACS) in the observation group were lower than that in the control group. There was statistical difference between the 2 groups (*P* < .05), for patients with colorectal cancer treated by infusion port.
Di Gu et al (2020) China^[30]^	Measure outcomes: Self-efficacy scale; Mental resilience scale; Treatment compliance-personalized regular medication execution record card; Evaluation of objective criteria for short-term efficacy of solid tumors; Time: pre-intervention and post-intervention	Feasibility: Recruitment rate, unspecified Complete rate: attrition rate 0% (intervention n = 48 usual n = 47) Acceptability: agreements: unspecified	Outcomes: After intervention, the total scores of role function, physical function, emotional function, cognitive function, social function and quality of life in the observation group were significantly higher than those in the control group, with statistical significance (*P* < .05).

Two systematic review authors, X.G. and X.S., independently screened all references to assess different constructs. In the case of differing evaluation results, the two authors will collaborate to analyze, discuss, and reconcile the findings.

### 2.4. Quality assessment

The Effective Public Health Practice Project (EPHPP) was used to assess the quality of the included studies.^[21]^ The tool was developed to assess the “quality of the evidence,” and appeared to measure different constructs, selection bias, design, confounders, blinding, data collection, and dropouts. The quality rating provide the evidence of a grade (weak, moderate, and strong) in Table 4.

**Table 4:** Quality assessment of the included studies (n = 11).

Author; (year); Country [Reference Number]	Selection Bias	Design	Confounders	Blinding	Data Collection	Dropouts	Quality Rating
Maryam Abedini et al (2019) Iran^[29]^	S	S	S	M	S	S	S
Nicole C Allard et al (2007) Canada^[32]^	S	S	S	W	M	S	M
Amy E. Richardson et al (2017) New Zealand^[26]^	W	S	S	S	S	S	M
Sandra Ward et al (2000) USA^[22]^	W	S	W	S	M	M	W
Heidi Scharf Donovan et al (2001) USA^[19]^	W	S	W	W	W	M	W
Nicole C Allard et al (2016) Canada^[27]^	M	S	S	S	M	S	S
Elizabeth A Grunfeld et al (2018) UK^[28]^	M	S	W	M	M	S	M
Kim Y et al (2002) USA^[23]^	M	S	S	S	M	S	S
Yanhong Yao et al (2020) China^[24]^	S	S	W	W	W	S	W
Yiping Yuan et al (2022) China^[25]^	S	S	W	W	M	S	M
Di Gu et al (2020) China^[30]^	S	S	W	W	M	S	M

Selection bias: Strong – very likely to be representative of the target population and greater than 80% participation rate; Moderate – somewhat likely to be representative of the target population and 60–79% participation rate; Weak – all other responses or not stated.

Design: Strong – RCT and CCT; Moderate – cohort analytic, case–control, cohort or an interrupted time series; Weak – all other designs or design not stated.

Confounders: Strong – controlled for at least 80% of confounders; Moderate – controlled for 60–79% of confounders; Weak – confounders not controlled for, or not stated.

Blinding: Strong – blinding of outcome assessor and study participants to intervention status and/or research question; Moderate – blinding of either outcome assessor or study participants; Weak – outcome assessor and study participants were aware of intervention status and/or research question.

Data collection methods: Strong – tools were valid and reliable; Moderate – tools were valid but reliability not described; Weak – no evidence of validity or reliability.

Dropouts: Strong – follow-up rate of > 80% of participants; Moderate – follow-up rate of 60–79% of participants; Weak – follow-up rate of < 60% of participants or withdrawals and dropouts not described.

Quality rating: S: strong; M: moderate; W: weak. Strong: If a study had no weak ratings and at least 4 strong ratings, then it was considered strong; Moderate: If the study had fewer than 4 strong ratings and one weak rating, it was rated moderate; Weak: If a study had two or more weak ratings, it was considered weak.

## 3. Results

### 3.1. Process of study selection

In an extensive search across 8 databases, a total of 1433 articles were initially identified. Subsequent manual screening and the utilization of EndNote 20 led to the exclusion of 750 duplicates. The titles and abstracts of the remaining 683 articles were meticulously evaluated against predefined inclusion and exclusion criteria. This process resulted in the exclusion of 577 articles, leaving 120 for in-depth full-text review. Ultimately, 11 studies met all criteria and were included in this review. Figure 1 provides an elaborate breakdown of this selection process. The primary reasons for exclusion encompassed: studies not focusing on cancer patients, lack of intervention-based research, interventions not aligning with the common-sense model, and the absence of full-text research.

### 3.2. Result of quality assessment

Table 4 delineates the quality assessment of the included articles. Within this assessment, 3 articles were adjudged as “strong” in terms of research quality. Another 5 articles were categorized at a “moderate” level, while 3 studies were deemed “weak.” A notable issue identified was selection bias, as evidenced by low recruitment rates in some articles. Despite all studies employing randomized control methods, several articles lacked clarity in their methodological descriptions.^[19,22–25]^ Furthermore, there was an omission of details regarding confounding factors in some studies. The issue of blinding – whether both evaluators and participants were blinded – remains unspecified in many of these studies. However, it is noteworthy that the overall follow-up rate in these studies was equal to or greater than 80%.

### 3.3. Characteristics of intervention

Research trials were completed in the USA (n = 3, 27.3%), China (n = 3, 27.3%), Canada (n = 2, 18.2%), the UK (n = 1, 9.1%), Iran (n = 1, 9.1%), and New Zealand (n = 1, 9.1%). Three of the studies were conducted before 2015, and 8 were conducted over the last 10 years. All studies were randomized and controlled. The randomization methods of design included a randomization table generated by computer software and an online randomization program. However, 5 studies did not include show the methods in the article (Table 1).

### 3.4. Characteristics of participants

The reviewed articles encompass a diverse range of cancer types. Six articles specifically addressed breast cancer, 3 were dedicated to colorectal cancer, 2 focused on prostate cancer, while others covered head and neck cancer, pancreatic cancer, intracranial tumors, and non-small cell lung cancer. Notably, only 2 articles^[19,22]^ investigated patients with metastatic or progressive cancer, with the remainder excluding metastatic cases. The stages of treatment among these studies varied widely, including newly diagnosed patients commencing surgery,^[22,24,26–28]^ as well as those undergoing radiation, chemotherapy, and combination therapies.^[19,22,23,25,26,28–30]^ The interventions were tailored to address specific needs based on the study’s focus, such as managing pain,^[19,22]^ mitigating radiation therapy side effects,^[23]^ enhancing sexual satisfaction,^[29]^ or facilitating a return to work.^[28]^ The size of the intervention groups in these studies ranged from 31 to 77 participants, with all patients being 18 years of age or older (as detailed in Table 1).

### 3.5. Theoretical framework of the interventions

Nine interventions used only the common-sense model and 2 implemented interventions using 2 different theories. A British paper used goal theory and CSM,^[28]^ and a Chinese paper combined the health-behavior model health education theory and CSM.^[24]^ Although there is no uniform year for the common-sense model cited by everyone, they all accurately express the core idea of the common-sense model (perception, emotion, and coping); however, the emphasis is different (Table 1).

### 3.6. Intervention content

#### 3.6.1. Content

The cornerstone of the Common-Sense Model (CSM) lies in interventions that pivot around patients’ perceptions of their disease.^[31]^ Our study encompassed cancer patients facing varied challenges at different stages of treatment, including chemotherapy, radiotherapy, and surgery. Consequently, the intervention content was meticulously tailored to align with the specific threats and disease perception statuses of patients at each treatment stage, with these threats being a key focus in outcome measurement. However, the actual measurement of disease perception is notably infrequent, with only 2 instances of such measurement reported.^[22,26]^ Additionally, we observed that the specific intervention content, formulated within the CSM framework, lacked consistency and uniformity. In some cases, interventions did not fully leverage the CSM framework. Emotional outcome indicators were only documented in a handful of studies.^[23–25,27]^ Moreover, the coping strategies incorporated within the intervention content were complex, posing challenges in effectively advancing the intervention content. In the following section, we provide a comprehensive summary of the complete interventions, as delineated in the 11 articles reviewed (Table 2):

(1) First, assess patient status before each intervention,^[26]^ and 3 Chinese interventions at different time periods also indicate an understanding of the current patient status.^[23–25,30]^ The patient is asked to respond to the current threat and rate its effectiveness.^[27,28]^ If there is a misunderstanding of their own status and threats and responses, they need to clearify the misunderstanding and fill in the information better in the next step.^[19]^ Literature shows that patients need to set goals after assessing their own condition and continue to self-regulate.^[25,28,29]^

(2) About the disease: For the 5 aspects of the disease, consequences (impact on life of symptoms), time (duration of treatment recovery), control (coping with the consequences of treatment or treatment and coping with emotions - concern, emotional dimension), identity (perception of symptoms), coherence (understanding disease).^[19,23–26,28,29]^ The countermeasures include: problem-centered solution; How to manage emotions related to diagnosis; Encourage positive emotions; Spouse support; Mindfulness and physical activity; Experience sharing with other patients; Support with family and community; Get information through brochures.^[22–24,26,28,29]^ As for threats such as: sexual satisfaction, difficulties in returning to work, postoperative symptoms, side effects of drugs after chemotherapy. Patients in treatment will face many problems, which should be one aspect of the difficulty in formulating intervention content. The response measures included symptom management, community support and family support.

(3) Patients express feelings and emotions^[22,24,26,27,30,32]^ or positive emotions.^[25,27–29]^ Although the model has an emotional correspondence with the disease, the measurement results corresponding to the intervention content are rarely measured.

(4) Understanding the patient’s future concerns such as recurrence.^[26,29]^ Explore the results of filling in the new information and responding to the old information, derive the expected benefits of the new action, and make patients more aware of the effectiveness of the intervention.^[19]^

#### 3.6.2. Dosage

The number of interventions was 2-5 times, 4 studies were 2-time interventions,^[22,23,27,32]^ 2 studies were 3-time interventions,^[26,29]^ and 4-time and 5-time intervention studies each had one paper.^[19,28]^ The intervention time ranged from 8 to 120 minutes. Three studies that needed to be conducted for more than 1 hour,^[26,28,29]^ and there were studies whose duration varied from 8 to 112min depending on the individual problem of the patients. The Chinese literature does not specify the time of each intervention or the total number of interventions, which is characterized by the intervention from the start of treatment to the duration of treatment to follow-up (Table 2).^[24,25,30]^

### 3.7. Delivery format

The characteristics of the study implementers were as follows: most were nurses (n = 5),^[19,22,25,27,30]^ there were 2 study team interventions (n = 2),^[28,32]^ and there were therapists, health psychologists, partners of study members and clinical members, and doctor-nurse associations (n = 4).^[23,24,26,29]^ There were 4 cases of face-to-face communication,^[19,23,26,29]^ 3 cases used only telephone communication,^[22,27,32]^ and 4 cases chose face-to-face communication first and then intervention through telephone, we-chat or toolkit.^[24,25,28,30]^ There were 6 additional recordings, toolkits, and manuals (Table 2).^[22–24,26,28,29]^

### 3.8. Intervention outcomes

Among the 11 studies, most did not measure all elements of the self-regulation common sense model, including threat, disease perception, coping, and outcome. This is an outcome measure of the focus of an intervention. No identical outcome measures were used to compare the intervention effects, and the measurement tools were not uniform. Some articles adopt a qualitative approach to collecting results such as intervention satisfaction, pain treatment satisfaction and job status.^[19,22,26,28]^ Therefore, the results were more heterogeneous (Table 3).

#### 3.8.1. Illness perception

Two studies measured this indicator in terms of disease perception. For patients with head and neck cancer who had a treatment plan, compared to the control group, patients who received the intervention had increased treatment control perceptions at 3 months (*P* = .01).^[26]^ For gynecological cancer patients with pain, all women reported a decrease in barriers between baseline and the 2-month follow-up (*P* < .05).^[22]^

Some studies have suggested using common sense model intervention to measure disease perception before and after intervention^[14]^ to identify disease outcomes improved by disease perception, namely CSM.

#### 3.8.2. Coping

From the perspective of self-management strategies, self-efficacy was measured,^[25,30]^ pain index was managed,^[22]^ and pain management was qualitatively investigated.^[19]^

There was a significant shift in women from unacceptable pain management at baseline to acceptable pain management at the 1-month post-test (*P* < .05). 32 (68%) reported that their pain was better managed and 27 (57%) were better able to manage the side effects of pain medication. For patients with colon cancer using intravenous infusion ports, the self-management Efficacy Scale (SUPPH) score was higher than that of the control group (*P* < .05). However, no approved measures of cognitive or emotional coping style have been seen reported. Beliefs regarding coping styles and actions must be identified.

#### 3.8.3. Disease outcomes

In terms of disease outcomes, sexual satisfaction, measurement of functional recovery, physical conditions such as pain sleep and urinary tract, emotional aspects such as sadness, rework conditions, medication treatment compliance, quality of life and satisfaction were measured as intervention outcomes. We obtained good results with this intervention.

Comparing scores at baseline and one month after the intervention, sexual satisfaction scores measured increased in the intervention group and decreased in the control group.^[29]^ For pain management, we obtained this outcome in 2 studies. All women reported a decrease in barriers between baseline and 2-month follow-up (*P* < .05); all subjects experienced a decrease in pain interference with life scores between baseline and 1-month post-test (*P* < .05); 40 (85%) said they were more confident using pain medication; and 38 (80%) indicated more confidence in talking with their physicians or nurses about pain.^[19,22]^ Patients who needed to return to work were followed up for 6 months after the intervention. 30% of the usual care group had returned to full-time or part-time work (including phased return to work) compared to 43% of the intervention group.^[28]^

The attentional focus and symptom management intervention (AFSMI) on functional status (home management disruption) showed a result at T2 (t=–1.71; *P* = .09) and at T3 (t=–0.65; *P* = .10), so there was no significant difference between the intervention group and the control group. Using paired sample t tests showed a significant decrease in home management scores for both intervention groups between T2 and T3 (t = 4.84; *P* < .01). Comparisons showed a significant difference between the experimental and control group at T2 (t=–2.20; *P* = .03) on emotional distress scores.^[27,32]^

For head and neck cancer in the treatment, compared with the control group, patients who received the intervention had an increased social quality of life at 6 months (*P* = .01). The intervention was particularly helpful for patients exhibiting distress at baseline.[3] For prostate cancer patients undergoing radiation therapy, subsequent t-test analyses revealed that patients in the comparison group reported more sleeping problems and fatigue than those in the intervention group.^[23]^ Nursing compliance, nursing satisfaction, and cognitive identification scale (LOTCA) and mental state test scale (MMSE) scores were higher in the observation group than in the control group. There was statistical difference between the 2 group (*P* < .05). The incidence of adverse reactions was lower in the observation group than in the control group among patients undergoing surgery for intracranial tumors. There was statistical difference between the 2 group (*P* < .05)^[24]^ The observation group after intervention hospital anxiety and depression scale (HADS), the scores of insomnia scale (AIS) and simplified fear of disease progression scale (FOP-Q-SF) in Chinese Cancer patients were lower than those in the control group. There was significant statistical difference (*P* < .01). The incidence of postoperative complications, financial problems, recurrence concerns, image problems and family stress in the cancer quality of life scale (QLACS) in the observation group were lower than those in the control group. There was significant statistical difference for patients with colorectal cancer treated by infusion port(*P* < .05).^[25]^ Non-small cell lung cancer chemotherapy patients after intervention, quality of life in the observation group was significantly higher than that in the control group. There was significant statistical difference (*P* < .05).^[30]^

### 3.9. Feasibility and acceptability

#### 3.9.1. Feasibility

In these 11 articles, the feasibility of the intervention was assessed using the recruitment and attrition rates. With a recruitment rate of 21% to 83%,^[22,23,26–28,32]^ there are many reasons for rejection, definitively declined participation, voiced an interest in participating “on another day,” don’t meet the study eligibility criteria. It is worth noting that the loss rate is low, except for 19% of patients with tumor pain who returned to work in the intervention study,^[22,28]^ the other is probably less than 15%. Studies have shown that short-term, low-cost telephone intervention and the time interval seemed reasonable (Table 3).^[32]^

#### 3.9.2. Acceptability

Because the intervention discusses questions and therapy, someone to listen to, learn coping strategies and someone suggests early intervention.^[26]^ 86% believed the information would be helpful to them, and talk more openly.^[22]^ They learned something new.^[19]^ The booklet was viewed as convenient and easily shared with others.^[28]^ Nursing compliance, nursing satisfaction, and scores were higher in the observation group than in the control group (Table 3).^[24]^

These studies show that research limitations are the greatest need for intervention (patients with a high degree of health threat are reluctant to participate in intervention), not exploring the basis for individual barriers, and long-term observation is needed.^[22]^ The content of the intervention must be specific.^[32]^ Measurement indicators must be measured objectively.^[27]^

## 4. Discussion

This review aimed to understand the impact of the common-sense model of self-regulation on cancer patients. The results of the study showed that the intervention of the self-regulating common sense model improved home management function, fatigue, response to surgery, depression, anxiety, and quality of life in cancer patients. This review includes specific content on CSM interventions to inform future research on the use of common-sense models of self-regulation in cancer patients.

The history of CSM is for more than 50 years. A study on visual atlas analysis of the literature on self-regulation common sense models shows that CSM was in the early stage of development, and researchers mainly focused on verifying and revising the applicability of this model in different diseases before 2011. From 2012 to 2015, CSM was in the experience summary stage, and researchers used the model to develop intervention programs; After 2016, CSM entered the summary and deepening stage, in which researchers conducted practical application, evaluation and integration, and used a variety of research methods to deeply explore the dynamics and integrity of the model.^[31]^ The 11 studies that we searched for interventions in cancer patients based on the self-regulating commonsense model had a gap of more than 10 years before 2015. The reasons for our analysis may be: First, the database we searched was insufficient, such as Scopus database, which we did not know about before. Second, we found that researchers are more inclined to explore the correlation between disease representation dimensions and health coping outcomes of cancer patients through the self-regulating common-sense model, or explore the determinants of disease behavior and outcomes. Observational studies and qualitative studies are more common. Third, there are interventional studies based on the common-sense model of self-regulation, which are common in educational interventions for diabetes, hypertension, and cancer screening for healthy people. Fourth, there are many factors that need to be considered in the investigation and implementation of intervention studies based on the common-sense model of self-regulation, and there are many more factors that need to be considered in cancer patients, such as the health threats of cancer patients.

Eleven articles were included in this review, and although 3 were rated as weak in quality, we analyzed all of them because of the small number of articles on CSM intervention in cancer patients. This suggests that more high-quality studies are needed to investigate the effects of CSM interventions in patients with cancer. In the study design, attention should be paid to difficulties in recruitment rates, explicit randomization methods, control for confounding factors, and blind methods for participants and evaluators.

From the study subjects, we can see that interventions are more common in breast cancer, among which two studies have caught our attention.^[19,28]^ The subjects included in these studies were patients with more than 3 types of cancer, but the number of the control intervention group was 26 to 61. It is necessary to explore the intervention effect of CSM in different cancer types.^[33]^ The importance of expert correspondence, teams of central scientists and medical care should be emphasized in the construction of intervention programs, because it is necessary to resolve patients’ misunderstandings of the disease.^[34]^ In the included studies, the intervention location was not specifically stated in the hospital or at home. Interventions with manuals, telephone interventions, or combinations of other elements are all effective in terms of outcomes, but it is not clear which form is more effective. Previous reviews have suggested that the conclusions of telephone interventions for managing symptoms in cancer patients are tentative.^[35]^ Previous studies have shown that noninvasive interventions for lung cancer have an effect on patients’ quality of life, and telephone education has an effect on pain and depression.^[36]^

The CSM theory is characterized by disease perception, disease cognition and emotion, and coping style. Now, there is no unified intervention content based on CSM for a particular disease. Some interventions have not completely applied the framework, and the coping methods for the intervention content are very complex. There are many coping styles and both positive and negative factors.^[37]^ Therefore, the content of intervention for cancer patients based on the common-sense model of self-regulation still needs to be explored. There are many threats in the course of cancer treatment, which not only have an impact on intervention content, but also have an impact on the intervention effect.^[38]^ As in the intervention studies we included, studies need to control for these conditions in terms of whether or not to biopsy,^[27,32]^ and working status and hormone therapy.^[23]^ In patients with cancer, family members may conceal the disease, whether to inform the patient or not, and whether to tell the patient the diagnosis will affect the disease perception intervention.^[39]^ In some cases, this is also a condition that needs to be controlled. We need to consider how interventions targeting only one prominent threat will be spelled out clearly, and how other threats can be better controlled.

According to the review, we sorted out the characteristics of current CSM interventions: the number of interventions was 2 to 5, each time 8 to 112 minutes, preferably depending on the patient’s condition. Merely a high frequency of interventions does not equate to their efficacy. These interventions encompass diverse methods, including direct face-to-face interactions, telephonic consultations, and the utilization of toolkits. Notably, 6 studies employed pamphlets or videotapes, methods that come highly recommended. Our analysis indicates that in 6 of the articles, follow-up assessments were conducted, suggesting that such follow-ups are more likely to reveal the tangible impacts of CSM-based interventions. Consequently, it is imperative to implement follow-up procedures post-intervention to accurately gauge and potentially enhance the outcomes.^[40,41]^

## 5. Conclusions

Interventions based on the common-sense model of self-regulation are currently implemented mainly in patients with breast cancer. This intervention has been associated with cancer patients undergoing surgery, radiotherapy, or chemotherapy, and has had a positive impact, improving compliance, quality of life, mood, or reducing symptoms. Illness perception can be considered as the main outcome measure. The intervention content is generally summarized in 4 points: first, the assessment and determination of the target to solve the problem; then, the knowledge education and psychological adjustment of the disease or problem; and finally, the feedback of the target and the discussion of future problems. More intervention studies should be conducted on the problems faced by cancer patients at different stages, so as to continuously summarize and improve the content of knowledge education and psychological problems.

## Author contributions

**Conceptualization:** Xue Gu.

**Data curation:** Xue Gu.

**Formal analysis:** Xia Shen, Jun-Rui Zhou, Jiang-Hui Chu.

**Funding acquisition:** Lei Jiang.

**Methodology:** Xue Gu.

**Project administration:** Lei Jiang.

**Supervision:** Lei Jiang.

**Visualization:** Xue Gu, Xia Shen, Jun-Rui Zhou, Jiang-Hui Chu.

**Writing – original draft:** Xue Gu.

**Writing – review & editing:** Xia Shen, Jun-Rui Zhou, Jiang-Hui Chu.
